# Reply to Ma and Wang: Reliability of various in vitro activity assays on SARS-CoV-2 main protease inhibitors

**DOI:** 10.1073/pnas.2024937118

**Published:** 2021-02-10

**Authors:** Zhe Li, Yuxi Lin, Yi-You Huang, Runduo Liu, Chang-Guo Zhan, Xin Wang, Hai-Bin Luo

**Affiliations:** ^a^Guangdong Provincial Key Laboratory of New Drug Design and Evaluation, School of Pharmaceutical Sciences, Sun Yat-Sen University, 510006 Guangzhou, People’s Republic of China;; ^b^Center for Innovative Marine Drug Screening & Evaluation, School of Medicine and Pharmacy, Ocean University of China, 266100 Qingdao, China;; ^c^School of Life Sciences, Lanzhou University, 734000 Lanzhou, China;; ^d^Molecular Modeling and Biopharmaceutical Center, College of Pharmacy, University of Kentucky, Lexington, KY 40536;; ^e^Department of Pharmaceutical Sciences, College of Pharmacy, University of Kentucky, Lexington, KY 40536;; ^f^Marine Biomedical Research Institute of Qingdao, 266100 Qingdao, China;; ^g^Key Laboratory of Tropical Biological Resources of Ministry of Education, School of Life and Pharmaceutical Sciences, Hainan University, 570228 Haikou, China

Ma and Wang (1) tested our recently reported severe acute respiratory syndrome coronavirus 2 (SARS-CoV-2) main protease (M^pro^) noncovalent inhibitors (2) using their in vitro assays, and they obtained negligible or much lower inhibitory activities compared to ours. Knowing the discrepancy, we first carefully rechecked our original experimental records, and we did not find any potential concern with our data that had been repeated by multiple coauthors. Notably, the fluorescence resonance energy transfer (FRET)-based enzymatic assay used by Ma and Wang (1) is different from our FRET-based enzymatic assay in the M^pro^ protein (discussed below) and the FRET substrate [longer than the more popularly used substrate (3, 4) utilized in our assay].

**Fig. 1.**
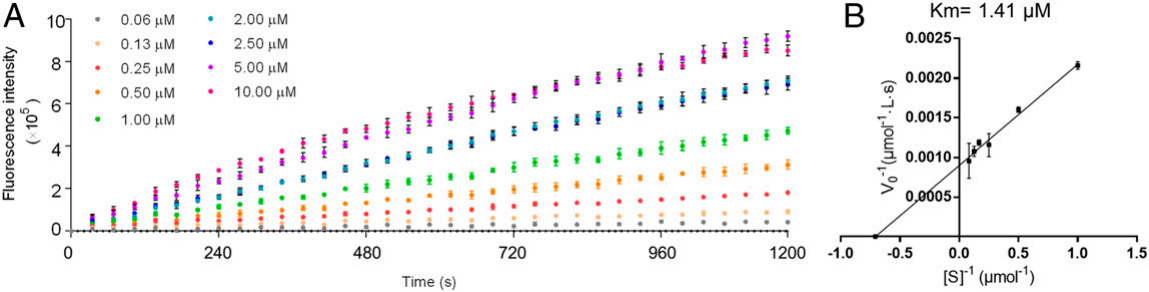
Michaelis–Menten kinetics of SARS-CoV-2 M^pro^ (100 nM) against substrate MCA-AVLQSGFR-Lys(Dnp)-Lys-NH2 at various concentrations. (*A*) Original data obtained. (*B*) Lineweaver–Burk plot used to determine the catalytic parameters.

Particularly for M^pro^, Ma and Wang (1) incorrectly state that a GST-tagged M^pro^ was used in our assay. Actually, as described in our paper (2), the GST tag was cleaved with thrombin; the GST tag was used only for conveniently isolating M^pro^ from the culture medium. So, the M^pro^ protein used in our assay is the true wild-type M^pro^ with native N and C termini. In comparison, the FRET-based enzymatic assay described by Ma et al. (5) used a C-terminal His-tagged M^pro^ protein. As noted correctly by Ma and Wang (1), M^pro^ requires a native N terminus to form the enzymatically active dimer. In fact, both the N and C termini of M^pro^ are very close to the active-site cavity in the dimer according to available X-ray crystal structures (Protein Data Bank [PDB] ID code 7BUY) (6), including one (PDB ID code 6WTT) shown by Ma et al. (5). Thus, an additional tag on the N or C terminus could interfere with M^pro^ binding with a ligand (substrate or inhibitor). So, a given ligand could have a lower binding affinity with the His-tagged M^pro^. In fact, we obtained Michaelis constant (*K*_m_) = 1.41 μM (Fig. 1) for the M^pro^ protein without any tag, and our reported catalytic efficiency (2) is close to the previously reported value (catalytic constant *k*_cat_/*K*_m_ = 28,500 M^−1^⋅s^−1^) (4). However, *K*_m_ = 28.2 μM for the His-tagged M^pro^ (5). So, the His-tagged M^pro^ has a ∼20-fold lower binding affinity with the substrate compared to the tag-free M^pro^. In other words, the activity determined by using the assay with a His-tagged M^pro^ might not reflect the actual activity with native M^pro^.

Ma and Wang (1) also used native mass spectrometry (MS) and thermal shift assays (TSA) to detect the protein–ligand binding. For binding driven by hydrophobic interaction the protein–ligand complex will most likely dissociate in MS (7). For TSA, false negatives are also known to occur (8, 9). Both assays might not be suitable for analyzing noncovalent inhibitors of M^pro^.

Finally, GC-376, a covalent inhibitor identified in their earlier reports, was used as a positive control to validate their assays by Ma and Wang (1). However, it is difficult to understand why their results show half-maximum inhibitory concentration (IC_50_) = 28 or 33 nM when the enzyme concentration was 100 nM. Their data, if validated, would imply that each GC-376 molecule inactivated multiple M^pro^ protein molecules through an unusual mechanism.
